# Molecular mechanisms underlying physical exercise-induced brain BDNF overproduction

**DOI:** 10.3389/fnmol.2023.1275924

**Published:** 2023-10-05

**Authors:** Marina Cefis, Remi Chaney, Julien Wirtz, Alexandre Méloux, Aurore Quirié, Clémence Leger, Anne Prigent-Tessier, Philippe Garnier

**Affiliations:** ^1^Département des Sciences de l’Activité Physique, Faculté des Sciences, Université du Québec à Montréal, Montreal, QC, Canada; ^2^INSERM UMR1093-CAPS, Université de Bourgogne, UFR des Sciences de Santé, Dijon, France; ^3^Département Génie Biologique, Institut Universitaire de Technologie, Dijon, France

**Keywords:** physical exercise, BDNF, exerkine, cognition, muscle, neuronal activity, cerebral blood flow, hemodynamic

## Abstract

Accumulating evidence supports that physical exercise (EX) is the most effective non-pharmacological strategy to improve brain health. EX prevents cognitive decline associated with age and decreases the risk of developing neurodegenerative diseases and psychiatric disorders. These positive effects of EX can be attributed to an increase in neurogenesis and neuroplastic processes, leading to learning and memory improvement. At the molecular level, there is a solid consensus to involve the neurotrophin brain-derived neurotrophic factor (BDNF) as the crucial molecule for positive EX effects on the brain. However, even though EX incontestably leads to beneficial processes through BDNF expression, cellular sources and molecular mechanisms underlying EX-induced cerebral BDNF overproduction are still being elucidated. In this context, the present review offers a summary of the different molecular mechanisms involved in brain’s response to EX, with a specific focus on BDNF. It aims to provide a cohesive overview of the three main mechanisms leading to EX-induced brain BDNF production: the neuronal-dependent overexpression, the elevation of cerebral blood flow (hemodynamic hypothesis), and the exerkine signaling emanating from peripheral tissues (humoral response). By shedding light on these intricate pathways, this review seeks to contribute to the ongoing elucidation of the relationship between EX and cerebral BDNF expression, offering valuable insights into the potential therapeutic implications for brain health enhancement.

## 1. Introduction

Physical exercise (EX) is the most efficient non-pharmacological strategy to improve health and prevent pathologies. Being physically active reduces not only the risk of developing cardiovascular, metabolic and chronic diseases but also brain disorders. Indeed, numerous studies reported the beneficial effects of EX across a lifespan on cognitive functions and neuroplastic mechanisms (Voss et al., 2011). EX reduces the prevalence and incidence of stroke, neurodegenerative diseases (e.g., Alzheimer’s, Parkinson’s diseases [AD, PD]) and mental disorders such as depression, schizophrenia and addiction (Dunn et al., 2005; Sofi et al., 2011; Paillard et al., 2015; Xu et al., 2023). Accumulating evidence supports that EX improves learning, memory, executive functions, attention in children (Chaddock et al., 2010; de Greeff et al., 2018) and adulthoods (Colcombe et al., 2006; Erickson et al., 2011; Sofi et al., 2011; Ludyga et al., 2020; Cheval et al., 2023). Moreover, EX has been linked to a decrease in stress and anxiety, as well as an improvement in emotional stability and sleep quality resulting in improved psychological well-being (Mandolesi et al., 2018).

In more details, the positive effects of EX on the brain may be explained by an increase in hippocampal neurogenesis, an enhancement of long-term potentiation (LTP) and the regulation of synaptic plasticity (van Praag et al., 1999; Farmer et al., 2004; Cefis et al., 2019). It has also been demonstrated that EX induces cerebral angiogenesis by increasing the density and sprouting of new capillaries from pre-existing vessels (Black et al., 1990; Ding et al., 2004). From animal and human studies, there is a consensus involving the neurotrophin brain-derived neurotrophic factor (BDNF) as responsible for the positive effects of EX on the brain. This neurotrophin is widely produced in the brain, where it plays a crucial role in neurogenesis, synaptic plasticity, angiogenesis and exerts neuroprotective effects (Korte et al., 1995; Bartoletti et al., 2002; Kim et al., 2004). The crucial role of BDNF is unquestionable since in animal studies, anti-BDNF strategies negate EX-associated cognitive benefits (Vaynman et al., 2003, 2004; Liu et al., 2008), while in humans, the val66met polymorphism (single nucleotide polymorphism in *bdnf* gene corresponding to a valine-to-methionine substitution), which is associated with a defect in activity-dependent regulated secretion, attenuates the cognitive advantage of EX (Egan et al., 2003; Erickson et al., 2012; Hopkins et al., 2012). The synergic interrelation between neuronal activity and synaptic plasticity designates BDNF as an ideal mediator of cellular and molecular mechanisms underlying cognitive and memory improvements induced by EX. However, even though BDNF involvement is incontestable, molecular mechanisms underlying EX-induced cerebral BDNF overproduction are not entirely delineated.

**Figure 1:**
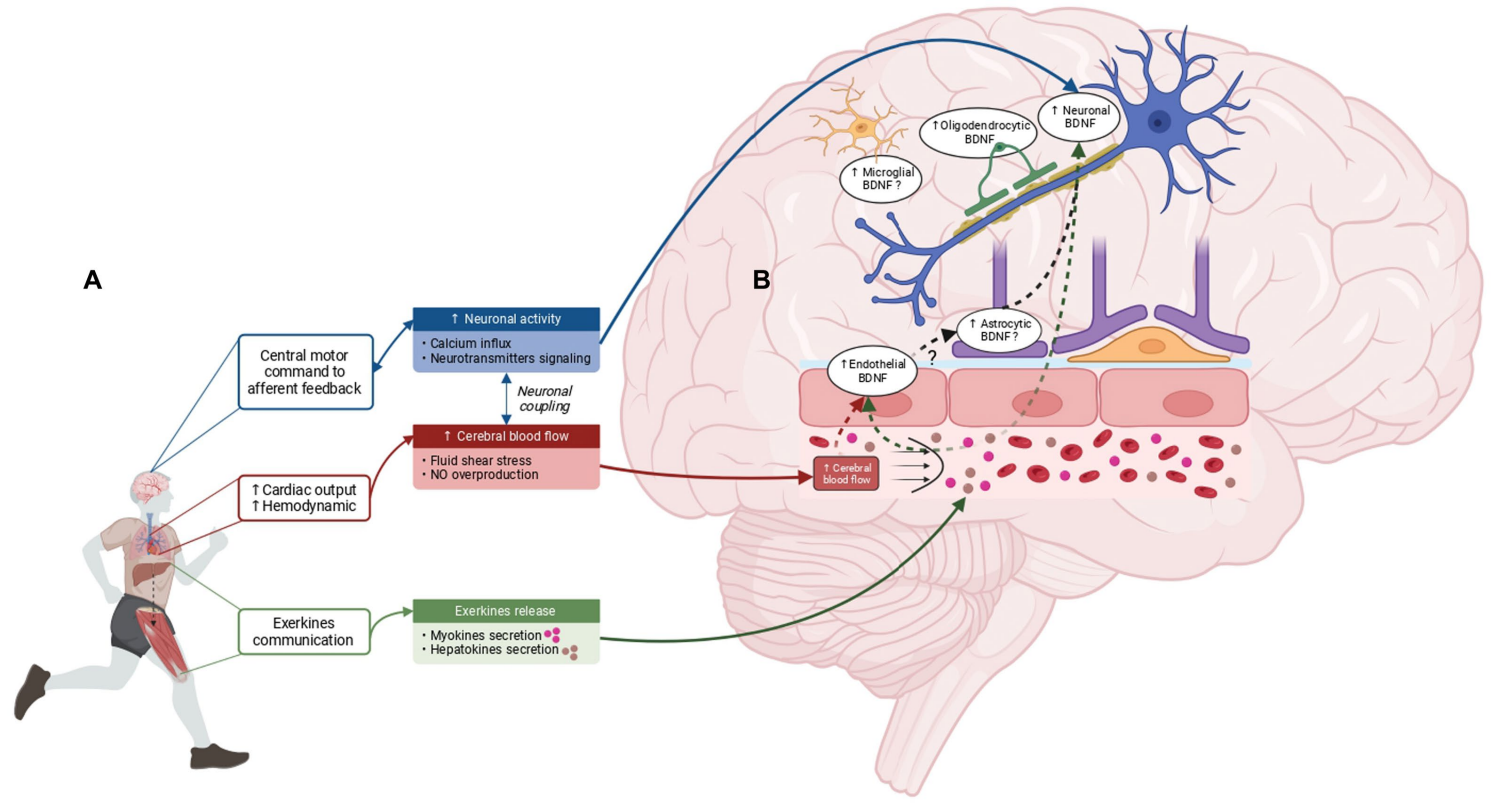
Mechanisms underlying EX-induced brain BDNF overproduction and cerebral BDNF-expressing cells in response to EX. **(A)** Cerebral BDNF increase in response to EX is thought to be driven by three main mechanisms: the increase in neuronal activity, the elevation of CBF and the release of exerkines from peripheral tissues. The brain detects EX through central motor control and afferent feedback which trigger an increase in neuronal activity involving neurotransmitter signaling and Ca^2+^ influx. In addition, EX leads to an increase in hemodynamic response subsequent to the cardiac output augmentation resulting in elevated cerebral blood flow and an increase in fluid shear stress. Of note, CBF elevation is also contingent upon neuronal coupling. Finally, peripheral tissues such as the liver and the skeletal muscle perceive EX and release exerkines into the bloodstream, capable of signaling to the brain and inducing cerebral BDNF increase. **(B)** BDNF overexpression in response to EX is not limited to neuronal cells. Studies indicate that BDNF overexpression is induced in endothelial cells and oligodendrocytes, while the involvement of microglia and astrocytes as potential sources of BDNF in response to EX requires further investigation. Created with BioRender.com.

In this context, this review aims to provide a comprehensive overview of scientific evidence on the different molecular mechanisms explaining how EX leads to cerebral BDNF upregulation. It is important to underscore that within the context of this review, the term “EX” has been utilized inclusively, encompassing a wide range of physical activities, irrespective of their specific type, duration, or intensity while these factors influence the cerebral expression of BDNF (Walsh et al., 2020; Fernandez-Rodriguez et al., 2022). After a brief description of BDNF metabolism and signaling pathways, this manuscript will focus on the different mechanisms responsible for EX-induced cerebral BDNF production. Hence, the increase in neuronal expression through activity-dependent mechanisms, the contribution of endothelial cells through cerebral blood flow (CBF) elevation (hemodynamic hypothesis) and the recent mechanisms involving humoral factors (exerkines) originating from peripheral tissues will be detailed and provided in Figure 1A. A better comprehension of the molecular mechanisms that lead to cerebral BDNF upregulation is required, not only because it could help to promote exercise prescription for brain health, but also since the modulation of these mechanisms could be an attractive possibility for the prevention and treatment of various brain pathologies.

## 2. BDNF: the key mediator of EX-induced cerebral plasticity

### 2.1. Localization and cellular expression

Initially discovered in the porcine brain by Barde et al. (1982), BDNF is a member of the neurotrophic factor family expressed by the neurons of the central nervous system (CNS) predominantly in the cortex and hippocampus (Ernfors et al., 1990; Hofer et al., 1990). In addition to neurons, many cells have been shown to express BDNF in the brain, such as astrocytes, microglia (Dougherty et al., 2000), pericytes (Ishitsuka et al., 2012) and endothelial cells (Leventhal et al., 1999; Nakahashi et al., 2000). Cerebral endothelial cells (CEC) are particularly noteworthy, as they synthesize 50 times more BDNF than primary cortical neuron cultures (Guo et al., 2008). Consistently, data reported that the *in vivo* removal of cerebral endothelium using a detergent (3-[(3-cholamidopropyl) dimethylammonio]-1-propane sulphonate, CHAPS), halved the cerebral BDNF content (Monnier et al., 2017b). Contrary to what its name may suggest, BDNF is also expressed in the cardiovascular system (heart, vessels) (Timmusk et al., 1993; Meuchel et al., 2011; Quirie et al., 2012; Prigent-Tessier et al., 2013), lungs, thymus, spleen (Aid et al., 2007), skeletal muscles (Cefis et al., 2022), and blood (Rosenfeld et al., 1995).

### 2.2. Metabolism and secretion

BDNF is initially synthesized in the endoplasmic reticulum as a precursor form, the pre-pro-BDNF that is subsequently transformed into pro-BDNF by the removal of the signal peptide. After N-terminal glycosylation of the N123 residue in the pro-domain, pro-BDNF is then either proteolytically cleaved intracellularly (i.e., by furin and pro-protein convertase 7 in Golgi but also by pro-protein convertase 1/3 in secretion vesicles) or extracellularly (i.e., by tissue plasminogen activator [tPA]/plasmin and matrix metalloproteinases) in mature BDNF (Mowla et al., 2001). Of note, extracellular processing of pro-BDNF is still a matter of debate since evidence came from *in vitro* studies using tagged pro-BDNF that do not reflect physiological conditions (Pang et al., 2004; Nagappan et al., 2009). BDNF is then secreted by either a constitutive or a regulated pathway, the latter involving the interaction of the pro-domain with the sortilin receptor (Chen et al., 2005). According to Aid and colleague’s data, *bdnf* gene consists in a common 3′ exon that encodes the entire pro-BDNF protein and at least eight 5′ non-coding exons (exons I-VIII). Each 5′ non-coding exon is spliced into the coding exon that encodes a similar BDNF protein product (Aid et al., 2007). Thus, the gene encoding for BDNF protein leads to multiple transcripts according to the use of alternative promoters and of the different splicing and polyadenylation sites. Although the significance of this complex organization is still obscure, it could afford for a distinct tissue-specific expression and regulation under specific physiological conditions (Timmusk et al., 1993; Aid et al., 2007). Concerning neuronal activity-dependent regulation of BDNF transcription, exons I and IV have been shown to be the most upregulated (Tao et al., 1998; Pruunsild et al., 2011; Zheng et al., 2011). Besides, promoters I- and IV-dependent *bdnf* transcription are both increased in response to EX (Intlekofer et al., 2013; Luft et al., 2022).

### 2.3. BDNF receptors and signaling pathways

BDNF acts through two different plasma membrane receptors, the tropomyosin-related kinase B (TrkB) receptor and pan75 neurotrophin receptor (p75^NTR^), the affinity being much higher for TrkB than p75^NTR^ (Chao, 2003) with its positive action being attributed to TrkB activation only. Conversely, the p75^NTR^ is the preferential receptor for pro-BDNF and its activation mainly leads to the activation of pro-apoptotic pathway (Friedman, 2000; Beattie et al., 2002). These data showed the yin and yang effects of this neurotrophin and highlighted the importance of the balance between the two forms for the proper functioning of BDNF (for review Lu et al., 2005). In neurons, TrkB receptors include the full-length (FL) form with a tyrosine kinase domain and the truncated forms (T1, T2, T3) devoid of the kinase domains (Stoilov et al., 2002; Fenner, 2012). All these isoforms share the same extracellular pattern and binding affinity for BDNF. The binding of BDNF to the TrkB-FL receptor induces its dimerization and auto-phosphorylation of different tyrosine residues at the cytoplasmic domain leading to the activation of three major signaling pathways involving the phospholipase Cγ (PLC-γ), the phosphatidylinositol-3 kinase (PI3K) and the mitogen-activated protein kinase (MAPK) pathways that trigger neurite outgrowth, cell differentiation, neuronal survival and synaptic plasticity (Huang and Reichardt, 2003; Reichardt, 2006; Fenner, 2012). For the major truncated form, it has been shown that when TrkB-FL and -T1 are co-expressed, TrkB-T1 can repress the TrkB-FL signaling (Fenner, 2012). Furthermore, BDNF binding to TrkB-T1 could induce both its own signalization (Rose et al., 2003), internalization and/or release (Alderson et al., 2000). Of note, also poorly documented, TrkB expression is not restricted to neurons but also expressed in different cells of the neurovascular unit such as endothelial cells (TrkB-FL and truncated) (Kim et al., 2004; Pedard et al., 2017) and astrocytes which in addition to p75^NTR^ (Rudge et al., 1994), expressed almost exclusively the truncated isoform, TrkB-T1 (Rose et al., 2003).

### 2.4. Cerebral cells overexpressing BDNF in response to EX

The cerebral cells responding to EX by an increase of cerebral BDNF expression are illustrated and summarized in Figure 1B.

#### 2.4.1. Neuronal expression

As an immediate-early gene, transcription of new BDNF mRNA occurs rapidly without the requirement of new protein synthesis in a process involving post-translational modification of pre-existing transcription factors. Using *in situ* hybridization, Neeper et al. (1995) were the first to report that voluntary wheel running increases BDNF mRNA in hippocampal and cortical neurons of rats. Since this initial finding, many studies have confirmed this discovery using different types, intensities and durations of EX (Oliff et al., 1998; Berchtold et al., 2005; Griffin et al., 2009; Cefis et al., 2019; Pedard et al., 2019). The mechanism regulating *bdnf* gene expression is intriguing since synaptic activation regulates the neuronal synthesis and secretion of BDNF, which in turn modifies synaptic morphology and efficacy (Poo, 2001). This review only focuses on biological mechanisms induced by EX that led to transcriptional upregulation.

#### 2.4.2. Endothelial expression

Initial investigations on endothelial BDNF expression have demonstrated its constitutive synthesis, release and regulation (Leventhal et al., 1999) by both peripheral (Nakahashi et al., 2000) and CEC (Bayas et al., 2002; Takeo et al., 2003) in culture. Secreted BDNF is bioactive since it exerts a neuroprotective effect by promoting neuronal growth and survival (Guo et al., 2008). *In vivo* studies have shown the presence of BDNF in the vascular endothelium both at peripheral and cerebral levels, with a significant part of cerebral BDNF corresponding to BDNF expressed by the cerebral endothelium (Monnier et al., 2017b; Cefis et al., 2020; Totoson et al., 2021). Moreover, endothelial BDNF expression was significantly higher after EX in the aorta, vein and cerebral microvessel fractions (Monnier et al., 2017a; Cefis et al., 2020). Conversely, pathologies associated with endothelial dysfunction, such as diabetes (Navaratna et al., 2011), cerebral ischemia (Bejot et al., 2011), high blood pressure (Monnier et al., 2017b) or rheumatoid arthritis (Pedard et al., 2017) decrease cerebral BDNF expression.

#### 2.4.3. Glial cells expression

The question of whether astrocytes serve as a source of BDNF in response to EX remains to be clarified. Research has demonstrated that EX stimulates astrocyte proliferation (Uda et al., 2006), as well as morphological changes in an AD mouse model (Rodriguez et al., 2013). In addition, studies have suggested that astrocytes overexpressing BDNF can promote hippocampal neurogenesis (Quesseveur et al., 2013) and their own remodeling (Holt et al., 2019). Besides, EX was reported to elongate astrocytic projections in the dentate gyrus and to increase TrkB expression in GFAP-positive cells (Fahimi et al., 2017) while in a PD mouse model, Palasz et al. (2019) showed that treadmill training protects neurons through increase in astrocyte-derived BDNF. Given the central role of these cells in maintaining CNS homeostasis, in modulating synaptic transmission and their function as gatekeepers of the blood–brain barrier (BBB) (Li et al., 2021), the observed astrocytic BDNF–TrkB expression following EX may reflect a complex interplay between endothelial and neuronal cells, with astrocytes acting as a bridge, as we propose in the hemodynamic hypothesis section. Further investigation is needed to unravel the intricacies of astrocytic involvement in EX-induced cerebral BDNF production.

In addition to astrocytes, EX has been reported to promote oligodendrogenesis (For review, Guo et al., 2020). After EX, oligodendrocyte proliferation has been observed in spinal cord and hippocampus of healthy mice (Krityakiarana et al., 2010; Matsumoto et al., 2011) and in various diseases models including multiple sclerosis (Guo et al., 2020) or chronic cerebral hypoperfusion (Jiang et al., 2017). In addition, oligodendrocytes modulate synaptic transmission through the secretion of BDNF (Jang et al., 2019). Consistently, glutamate transmission and vesicular release were decreased in a model of mice with conditional deletion for BDNF in oligodendrocytes while these effects were offsetted with the application of BDNF or TrkB agonist (7,8-DHF). Taken together, these data suggest that oligodendrocytes are involved as a cellular source in EX-induced cerebral BDNF expression.

Concerning microglial cells, EX has been shown to modulate microglial activation. Thus, Mee-Inta et al. (2019) have highlighted the regulatory effect of EX on microglial activation, leading to an increase in anti-inflammatory factors and a decrease in pro-inflammatory factors. Additionally, in a mouse model of AD submitted to long-term EX, microglial activation was decreased in cerebral cortex and hippocampus, accompanied by an increase of BDNF-positive cells (Xiong et al., 2015). While there is evidence suggesting that microglia can produce BDNF (Madinier et al., 2009) and that microglia promote learning and memory through BDNF signaling (Parkhurst et al., 2013), a recent article suggests that BDNF expressed by microglia is not produced in sufficient amount to modulate neuronal function (Honey et al., 2022). Therefore, further investigations are needed to fully elucidate the involvement of microglia in the cellular expression of BDNF in response to EX.

## 3. Cerebral mechanisms involved in EX-increased BDNF production

**Figure 2:**
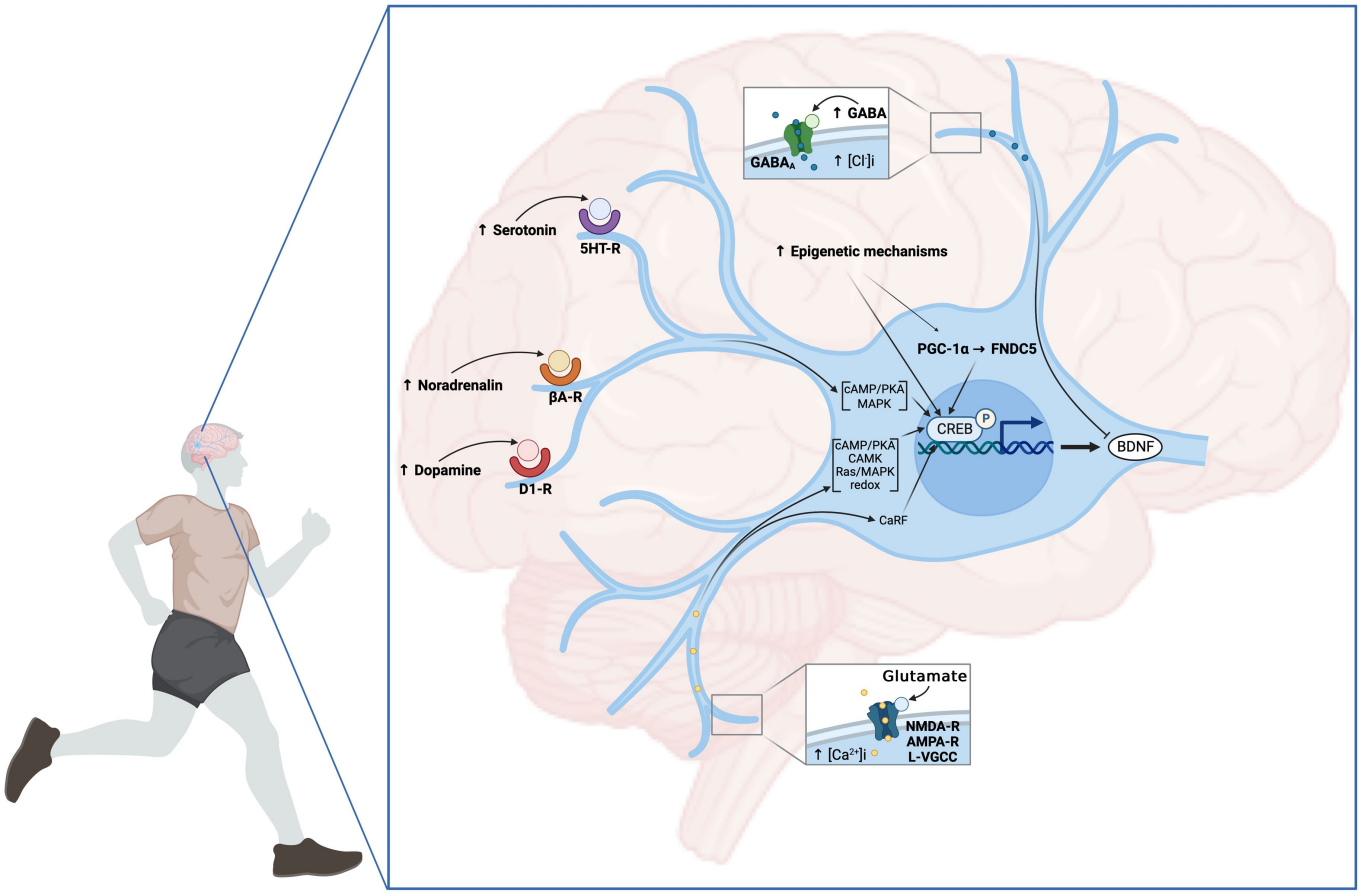
Neuronal mechanisms involved in EX-induced BDNF increase. From the central motor control to the afferent feedback, EX induces an increase in neuronal activity and the release of neurotransmitters leading to the upregulation of *bdnf* gene expression. The modulation of neuronal activity is primarily promoted by the elevation of Ca^2+^ influx through membrane L-VGCC. In addition to membrane depolarization, glutamatergic transmission mediated by AMPA and NMDA receptors plays a preponderant role in this process. Additionally, signaling from dopaminergic, noradrenergic and serotoninergic transmitters contributes to the upregulation of neuronal *bdnf* expression through D1-like receptor (D1-R), βadrenergic receptor (βA-R), and serotonin receptor (5HT-R), respectively. Conversely, GABA negatively affects hippocampal BDNF expression via GABA_A_ receptors. Multiple Ca^2+^-dependent mechanisms have been proposed as responsible for CREB phosphorylation including Ca^2+^-sensitive adenylate cyclase/PKA, Ca^2+^/CAMK, Ras/MAPK and the redox signaling. Another mechanism leading to neuronal BDNF expression is associated with the activation of neuronal PGC-1α/FNDC5/Irisin pathway. Finally, epigenetics mechanisms involving HAT, DNMT, HDAC activities and miRNA, particularly mi138 were demonstrated. Created with BioRender.com.

All the mechanisms described in sections a-e are summarized in Figure 2.

### 3.1. Calcium influx-induced neuronal activity

Neuronal activity-regulated gene expression has been proposed to be a key mediator for experience-dependent changes in the nervous system (Katz and Shatz, 1996). From initial studies showing that EX, from the central motor control to the afferent feedback, induced electrical activity and sustained hippocampal activation (Vanderwolf, 1969; Czurko et al., 1999), the mechanism by which EX induces neuronal BDNF synthesis has been well investigated. From electrical to chemical level, Ca^2+^ influx is critical in transmitting depolarization status and triggering synaptic activity. Thus, using physiologically relevant stimulation patterns of hippocampal neurons, Balkowiec and Katz (2002) showed that BDNF production needed not only Ca^2+^ influx but also mobilization from intracellular Ca^2+^ stores. Once initiated, Ca^2+^-dependent signaling cascades trigger activity-induced changes in gene expression and protein synthesis (Ghosh and Greenberg, 1995). Using the induction of BDNF mRNA, West et al. (2001) have shown how Ca^2+^ induces neuronal gene expression. Membrane depolarization and neurotransmitter binding lead to ligand-gated and voltage-gated Ca^2+^ channels (L-VGCC) at the cell membrane, triggering the influx of extracellular Ca^2+^ into the cell. In terms of ligand-gated channels responsible for Ca^2+^ entry into neurons after EX, different mechanisms have been proposed involving preponderantly glutamatergic transmission through α-amino-3-hydroxy-5-methyl-4-isoxazolepropionic acid (AMPA) and *N*-methyl-d-aspartate-type (NMDA) receptors (Kitamura et al., 2003; Vaynman et al., 2003; Nishijima et al., 2012). As stated above, promoter IV-dependent *bdnf* transcription, one of the main promoters driving neuronal activity-dependent BDNF expression, is the most thoroughly investigated (Tao et al., 2002). Using a mouse model with impaired activity-dependent *bdnf* expression through mutagenesis, Hong et al. (2008) revealed the existence of three Ca^2+^-responsive elements (CaREs) able to drive activity-dependent induction of promoter IV. Among the three DNA sequences that are cooperatively recruited for Ca^2+^-induced *bdnf* promoter IV expression, Tao et al. (2002) have shown that Ca^2+^-response factor (CaRF) is a regulator of activity-dependent *bdnf gene* expression through CaRE1 and that the transcriptional activity of CaRF is dependent on Ca^2+^ influx in a neuron selective manner. CaRE3 has also received specific attention. Indeed, once bounds by CREB (cAMP response element-binding protein), this latter is phosphorylated on serine 133 (Ma et al., 2014) and then induces BDNF transcription (Tao et al., 1998; Shen et al., 2001). Multiple Ca^2+^-dependent mechanisms have been proposed to be responsible for CREB phosphorylation at the upstream steps of CREB activation. These mechanisms included the Ca^2+^-sensitive adenylate cyclase/PKA (Shen et al., 2001), the Ca^2+^/calmodulin-activated kinase (CAMK) (Sun et al., 1994; Bito et al., 1996), Ras/MAPK (Wu et al., 2001) and the redox signaling (Radak et al., 2016) systems. Since BDNF has been shown to subsequently interact with NMDA receptors, CAMK and MAPK (Vaynman et al., 2003; Nakata and Nakamura, 2007), EX-induced changes in neuronal activity-dependent gene induction may use BDNF itself as a perpetuating loop.

### 3.2. Other neurotransmitter signaling

In addition to glutamate, signaling from cholinergic, noradrenergic and serotoninergic transmitters are involved in cerebral *bdnf* gene expression (Garcia et al., 2003; Ivy et al., 2003; Chen et al., 2007). The cortical acetylcholine (ACh) content is increased in rats following a short period of walking, suggesting that ACh is released even at low-intensity of EX (Kurosawa et al., 1993). Conversely, cholinergic transmission impairment is associated with a downregulation of the hippocampal BDNF pathway in rats submitted to Morris Water Maze training that could be reversed by galantamine, a selective ACh esterase inhibitor (Gil-Bea et al., 2011). Interestingly, the blockade of noradrenergic signaling blunts the EX-effect on *bdnf* gene transcription (Garcia et al., 2003; Ivy et al., 2003) and is reversed when animals received reboxetine, a selective noradrenalin reuptake inhibitor (Russo-Neustadt et al., 2004). Mechanistically, the binding of noradrenalin to adrenergic receptor activates the phosphorylation cascades and induces BDNF expression in hippocampal neurons (Chen et al., 2007). Dopamine (DA) has also been reported to be upregulated by EX and to induce an increase in serum Ca^2+^ levels, which can enhance DA synthesis in the brain (Sutoo and Akiyama, 2003). There is evidence showing that DA receptor activation leads to BDNF expression probably through D1-like receptor activation (Kuppers and Beyer, 2001; Williams and Undieh, 2009). In addition, serotonin (5-HT) signaling may also be involved in EX-induced *bdnf* expression but to a lesser extent since 5-HT2A/C blockade minimally altered EX-induced BDNF mRNA activation (Ivy et al., 2003; Russo-Neustadt et al., 2004). Finally, gamma-aminobutyric acid (GABA), the primary inhibitory neurotransmitter of the CNS, has been shown to decrease hippocampal BDNF expression and to impair learning and memory (Kim et al., 2012) while bicuculline, a GABA_A_ receptor antagonist, enhances memory consolidation by increasing hippocampal BDNF levels (Kim et al., 2012). Interestingly, the anxiolytic effect of a 3-week running protocol in mice was blocked by the infusion of bicuculline in the ventral hippocampus. These observed results suggest that GABAergic transmission in the hippocampus, in response to EX, may play an important role in dampening excitatory circuits that might otherwise trigger an anxious response. These findings demonstrate a complex and finely tuned adaptation (Schoenfeld et al., 2013).

### 3.3. Cerebral PGC-1α/FNDC5/irisin pathway

Primarily discovered as a secreted factor from skeletal muscle, irisin is regulated by the peroxisome proliferator activator receptor γ coactivator-1α (PGC-1α) pathway, cleaved from fibronectin type III domain-containing protein 5 (FNDC5) and released in blood circulation (Bostrom et al., 2012). In addition to its peripheral expression, FNDC5/irisin is also produced in different brain regions (Dun et al., 2013) and Wrann et al. (2013) were the first showing that EX increases hippocampal BDNF expression through the PGC-1α/FNDC5 pathway. Indeed, the authors reported that the knockdown of PGC-1α reduced FNDC5 expression in the brain. Using primary cortical neurons, forced expression of FNDC5 increases BDNF levels whereas RNA interference directed against FNDC5 reduces its expression. Consistent with these findings, another study in mice has reported that the val66met polymorphism is associated with reduced expression of brain FNDC5 and BDNF following EX (Ieraci et al., 2016). Additionally, in an AD mouse model, hippocampal neurogenesis and cognition are improved by EX through both FNDC5 and BDNF upregulation (Choi et al., 2018). Interestingly, data reported that the exposition of *ex vivo* human cortical slices to recombinant irisin leads to the activation of the cAMP–PKA–CREB memory pathway (Lourenco et al., 2019).

### 3.4. Epigenetic mechanisms

Among epigenetic mechanisms, DNA methylation and histone modifications through methylation or acetylation are the most studied processes affecting gene expression. Numerous studies have shown that EX can act as an epigenetic modulator of brain plasticity and cognition influencing the activity of enzymes responsible for methylation (DNA methyltransferase, DNMT), histone acetylation (histone acetyltransferase, HAT) or deacetylation (histone deacetylase, HDAC). Thus, various studies have demonstrated that different models of EX lead to the modulation of HAT, DNMT, and HDAC activities at BDNF promoters (Gomez-Pinilla et al., 2011; Intlekofer et al., 2013; Sleiman et al., 2016). These changes ultimately result in an increase in *bdnf* gene transcription associated with cognitive improvement. For example, the research conducted by Gomez-Pinilla et al. (2011) demonstrated that EX has the capability to decrease methylation of CpG sites. This reduction occurs through the dissociation of methyl-CpG-binding protein 2 due to its phosphorylation, leading to enhanced *bdnf* transcription (Gomez-Pinilla et al., 2011). Additionally, Ionescu-Tucker et al. (2021) have recently highlighted that EX counteracts with the repressive histone modification trimethylated histone 3, lysine 9 (H3K9me3) at *bdnf* promoter, increasing BDNF expression in aged mice. Additionally, evidence suggests the role of small non-coding mRNAs on transcriptional gene silencing. Using transgenic mice in which Dicer which plays a pivotal role in the initiation of RNA silencing was inactivated, the overall decrease in hippocampal miRNAs was associated with a higher BDNF level leading to learning and memory improvements (Konopka et al., 2010). Using profiling array data, the authors identified two miRNAs (miRNA-138 and miRNA-384-5p) that potentially target BDNF mRNA. miRNA-138 was shown to act specifically on sirtuin 1 (SIRT-1), which regulates the acetylation status of PGC-1α responsible for *bdnf* gene expression through FNDC5 as stated in the preceding paragraph (Li et al., 2020). On the other hand, it has been reported that EX increases hippocampal SIRT-1 activity that downregulated miRNA-134 known to repress the translation of CREB and consequently that of BDNF (Gao et al., 2010). Other miRNAs have been proposed as regulators of BDNF (Varendi et al., 2014) and it has been reported that EX modifies the levels of many miRNAs in the brain (Cosin-Tomas et al., 2014; Pan-Vazquez et al., 2015). However, whether EX modulates miRNAs that directly affect cerebral *bdnf* gene expression requires further clarification.

### 3.5. Hemodynamic hypothesis: involvement of cerebral blood flow

EX is known to increase CBF in sensorimotor region and areas involved in cognitive capacities such as the prefrontal cortex, hippocampus and amygdala (Nishijima and Soya, 2006; Endo et al., 2013; Matsukawa et al., 2015). Considering the established synthesis of tPA by endothelial cells and its release into the bloodstream in response to different stimuli, including EX (Ding et al., 2011), a hypothetical mechanism can be proposed. Specifically, the elevation of CBF and the subsequent increase in fluid shear stress (FSS) induced by EX may result in an increase in tPA activity, which could then influence BDNF processing by cleaving pro-BDNF into its mature form through the conversion of plasminogen into active protease plasmin. Alternatively, tPA by facilitating neuronal NMDA receptors activation (Nicole et al., 2001; Anfray et al., 2020), could trigger Ca2+ influx and *bdnf* gene expression as stated in a precedent section. This notion is supported by the findings of Ding, Ying, and Gomez-Pinilla et al. (2011), who demonstrated that tPA-blocking strategies effectively abolished the increase in both pro- and mature BDNF in rat hippocampus, as well as the effect of EX on TrkB signaling and synaptic plasticity. The hypothesis that tPA produced by endothelial cells in response to FSS-related EX plays a key role in BDNF processing is plausible, particularly in light of evidence showing that blood tPA can cross the BBB via low-density lipoprotein receptor-related protein (LRP)-mediated transcytosis (Benchenane et al., 2005). Furthermore, considering the expression of NMDA and LRP receptors by endothelial cells (Hogan-Cann et al., 2019; Lu et al., 2019; Anfray et al., 2020), it is conceivable to postulate that tPA may function as an autocrine molecule, triggering the production of BDNF within these cells. To investigate these different hypotheses, the implementation of an endothelial tPA knockout (KO) model subjected to EX could provide valuable insights.

Strong evidence in favor of the hemodynamic hypothesis is also provided when strategies to reduce CBF were applied. One-week daily EX in rats with unilateral carotid occlusion (irreversible clamping) did not increase cortical BDNF levels in ipsilateral compared to exercised rats without carotid occlusion (Banoujaafar et al., 2014). Mechanistically, EX induces elevation of CBF, increases FSS which in turn stimulates endothelial nitric oxide synthase (eNOS) and the subsequent production of endothelial NO (Rubanyi et al., 1986). Interestingly, pharmacological inhibition of NO production by L-NAME (N-nitro-L-arginine methyl ester) (Chen et al., 2006) prevents the enhancement of BDNF mRNA occurring with EX while genetic deletion of eNOS abolishes the positive effects of EX on ischemic stroke (Endres et al., 2003). Accordingly, using an FSS-dependent increase in endothelial NO production, data demonstrate in Human umbilical vein endothelial cells subjected to different flows that endothelial BDNF expression was proportional to shear stress intensity (Prigent-Tessier et al., 2013). The hypothesis of NO regulation is also supported by a strong correlation, in cerebral microvessels, between BDNF protein levels and the activated form of eNOS (p-eNOS) phosphorylated at serine 1,177. Additionally, a positive association was also found between the elevation of p-eNOS and p-TrkB activation, in response to EX (Cefis et al., 2020). Besides, Monnier et al. (2017b) have shown that exposure of cerebral microvessel-enriched fractions or hippocampus sections from spontaneous hypertensive rats (SHR) to slow-releasing NO donor (glycerol trinitrate) increased endothelial BDNF production. Importantly, FSS-related EX-dependent response was prevented by TrkB inhibition (Wang et al., 2018). Collectively, these data and the one showing that NO controls t-PA release by human endothelial cells (Giannarelli et al., 2007) provide strong arguments suggesting that NO may be the intermediate between FSS and endothelial BDNF synthesis and maturation at the cerebral level.

**Figure 3:**
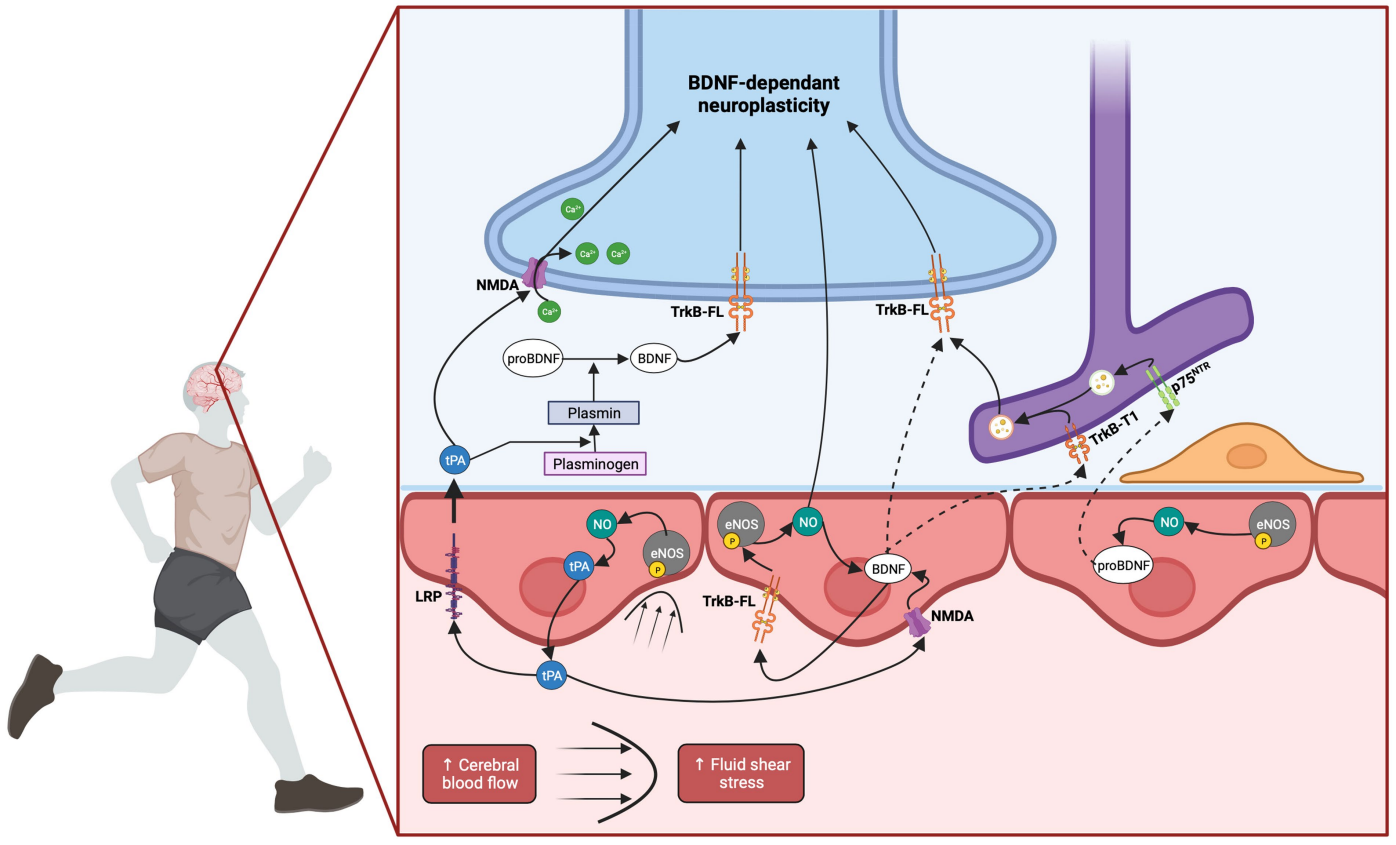
Hemodynamic hypothesis: involvement of cerebral blood flow in EX-induced BDNF increase. EX induces an increase in CBF, leading to elevated FSS. This mechanical stimulation triggers the activation of eNOS through its phosphorylation, resulting in the production of NO. The released NO has the potential to diffuse from endothelial cells to neurons, initiating neuronal BDNF expression. Additionally, endothelial NO has been demonstrated to induce BDNF expression in endothelial cells themselves. Endothelium-derived BDNF may directly bind to neuronal TrkB-FL receptors thus activating neuroplastic pathways. The proximity between synapses and capillaries supports this hypothesis. Alternatively, endothelium-derived BDNF could act in an autocrine manner, amplifying the NO response through endothelial TrkB-FL receptors. In parallel, astrocytes, through their nearby end-feet, might internalize either BDNF or pro-BDNF through their TrkB-T1 or p75^NTR^ receptors, respectively. Subsequently, they could re-secrete BDNF which might be transferred to neighboring neurons. Finally, the elevation of CBF and subsequent NO production could trigger tPA release. tPA has the potential to cross the BBB via LRP-mediated transcytosis. It could then facilitate neuronal NMDA receptor activation or influence the processing of pro-BDNF to BDNF, through the conversion of plasminogen into plasmin. Alternatively, tPA might facilitate endothelial NMDA receptor activation which could potentially contribute to endothelial BDNF expression. Created with BioRender.com.

Finally, the precise role of endothelial BDNF in the brain remains speculative, but there is compelling evidence suggesting its involvement in cerebral vessel vasodilation (Santhanam et al., 2010; Bordy et al., 2020), cerebral angiogenesis (Kim et al., 2004) and neuroplastic processes (Marie et al., 2018). The presence of endothelial TrkB-FL receptors suggests that endothelial BDNF may exert an autocrine effect by promoting NO production, which could then diffuse from endothelial cells to neurons, enhancing LTP and neuroplasticity (Hopper and Garthwaite, 2006). Alternatively, endothelium-derived BDNF may directly bind to neuronal TrkB-FL receptors and trigger neuroplastic pathways. The proximity between synapses and capillaries supports this hypothesis, although it still needs to be demonstrated. Finally, as mentioned above, endothelial BDNF may promote neuroplasticity through an astrocyte-dependent mechanism. Astrocytes have been implicated in the recycling of extracellular BDNF proteins, suggesting their involvement in facilitating BDNF availability. Two potential mechanisms can be proposed in this regard. A first mechanism may involve internalization of pro-BDNF through p75^NTR^ -dependent endocytosis which is subsequently re-secreted by astrocytes in its mature form (Bergami et al., 2008; Vignoli et al., 2016). The second mechanism implies BDNF release by CEC into the perivascular space. The nearby astrocytes end-feet able to internalize it through TrkB-T1 receptor could then transfer BDNF to neighboring neurons via transcytosis or a related mechanism. Recent research by Han et al. (2021) showing in astrocyte recycling, the re-secretion of TrkB-endocytic BDNF substantiates this theory. Consequently, the released BDNF from astrocytes into the extracellular space could bind to neuronal TrkB-FL triggering neuroplastic signaling pathway. Ultimately, the utilization of molecular strategies aimed at specifically inhibiting endothelial BDNF could serve as a potent tool to elucidate its precise role, especially in the context of neuroplasticity. All mechanisms proposed in the present section are summarized in Figure 3.

## 4. Peripheral mechanisms involved in EX-induced BDNF increase

**Figure 4:**
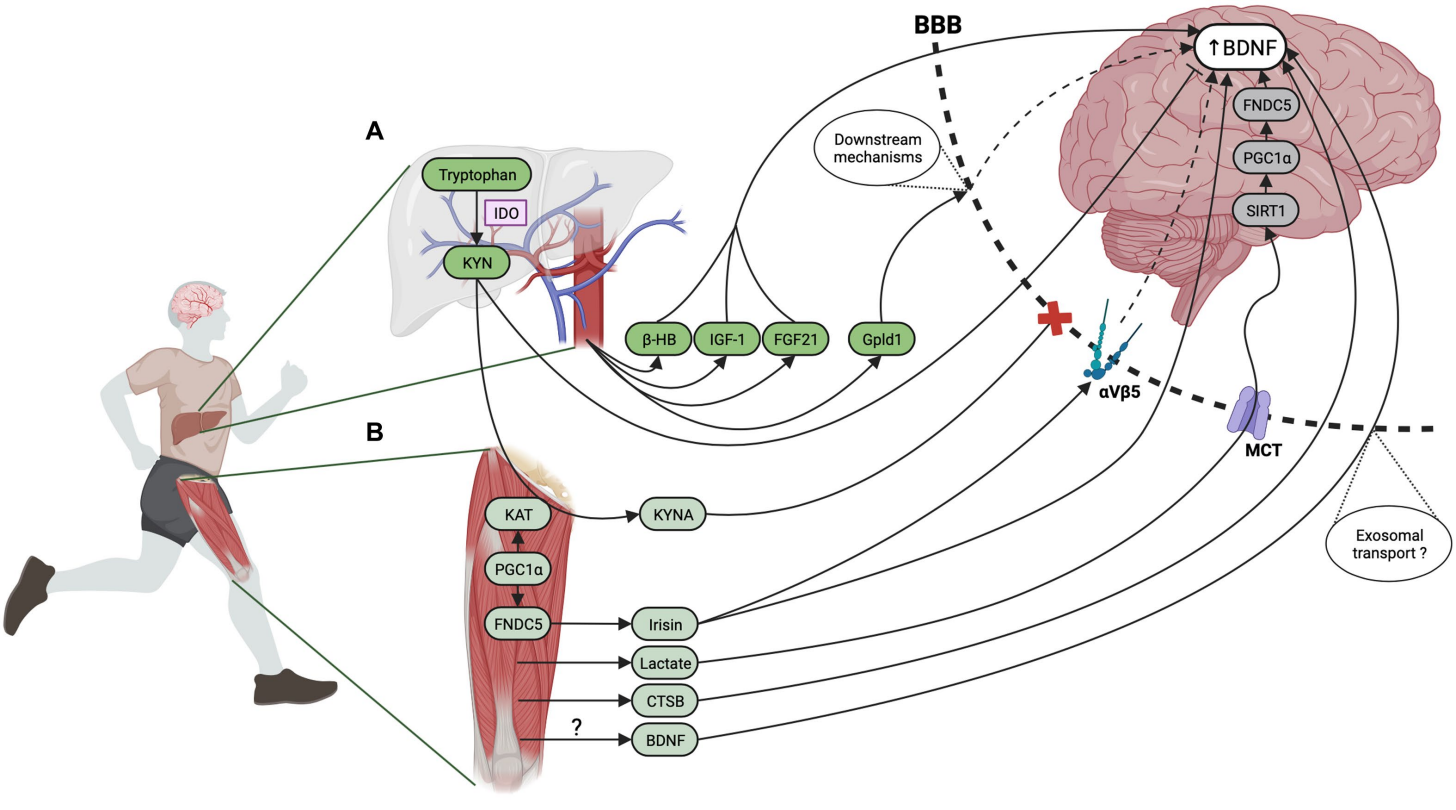
Peripheral mechanisms involved in EX-induced BDNF increase. Many organs respond to EX through the release of exerkines. Among them, the liver and the skeletal muscle have received particular attention and several exerkines originating from these organs have the potential to influence brain BDNF expression. **(A)** In response to EX, the liver secretes exerkines named hepatokines. Studies have reported that β-HB can cross the BBB and function as a signaling molecule promoting hippocampal BDNF expression by HDAC inhibition. In addition, IGF-1 can cross the BBB through IGF-1 receptor binding. Inhibition of IGF-1 signaling through blocking antibodies prevented EX-induced hippocampal BDNF expression. FGF-21 is also secreted by the liver in response to EX, can enter the brain and improve cognition possibly through an increase in brain BDNF expression since intraperitoneal administration of FGF-21 was associated with BDNF upregulation in aged mice. Recent research has unveiled Gpld1 as a newly discovered hepatokine that appears to correlate with cognitive performance in mice undergoing EX. Moreover, overexpression of Gpld1 in the liver has been associated with a significant rise in hippocampal BDNF expression and an enhancement of neurogenesis. Although Gpld1 does not cross the BBB, data suggest that this enzyme may be involved in coagulation as well as in the complement system cascades of molecules downstream of GPI-anchored substrate. **(B)** Several molecules named myokines are secreted by skeletal muscles in response to EX and have been shown to promote brain BDNF expression. Lactate that is significantly produced and released during EX can cross the BBB via MCT transporters. The pharmacological blockade of MCT in mice submitted to EX abolished hippocampal *bdnf* gene expression while intraperitoneal administration of lactate elicited a hippocampal BDNF increase comparable to that observed in trained mice. Lactate effect on hippocampal *bdnf* gene expression is thought to be dependent on the potentiation of NMDA glutamatergic transmission and upregulation of SIRT-1 activity fostering the PGC-1α/FNDC5/BDNF pathway. The myokine CTSB is also released by skeletal muscle during EX though an AMPK-dependent mechanism. *In vivo* experiments provide evidence that CTSB can cross the BBB and elicit BDNF expression while EX in CTSB KO mice failed to enhance neurogenesis and improve spatial memory. Although the role of skeletal muscle as a source of blood BDNF remains a topic of debate, recent studies using NMES as a model of muscle contraction seem to support this hypothesis. The BBB crossing of circulating BDNF is uncertain but exosomal transport might be involved. Additionally, peripheral delivery of BDNF has been shown to induce neurogenesis and increase hippocampal BDNF levels. During muscle contraction, the increased calcium signaling enhances PGC-1α activation which leads to an increase in FNDC5, a transmembrane protein that is cleaved during EX and released in the bloodstream as irisin. Data reported that irisin could cross or signal at the BBB, potentially via its recently discovered binding to activated integrin αVβ5 receptors. In an AD mice model, peripheral delivery of FNDC5/irisin rescued memory impairment and synaptic plasticity deficits through mechanisms dependent on cerebral BDNF. Conversely, the blockade of peripheral FNDC5/irisin attenuates the effect of EX on LTP and memory tests. PGC-1α activation during EX also stimulates the expression of KAT enzymes within skeletal muscle. This enzyme catalyzes the conversion of KYN, a neurotoxic metabolite that can cross the BBB and lead to depression to KYNA which is unable to cross the BBB. Mice with muscle-specific overexpression of PGC-1α were resilient to stress-induced depression and did not display decreased hippocampal *bdnf* gene expression while peripheral KYN administration induced depressive behavior in wild-type but not in transgenic animals. This last mechanism illustrates the crosstalk between peripheral organs, as KYN is a metabolite of tryptophan produced in the liver. Created with BioRender.com.

In the present section, our focus lies on elucidating the various humoral factors that have the potential to modulate brain BDNF levels in the context of liver- or muscle-brain crosstalk after EX. All mechanisms described in the following sections are summarized in Figure 4. We have assigned these factors into liver- or muscle-brain sections based on preliminary findings and their prominent expression in these organs. However, it should be emphasized that several molecules which will be expounded upon herein are known to be produced and secreted by various sources including the brain. In addition, while we have focused on molecules produced by the liver (Figure 4A) and skeletal muscle (Figure 4B), it should be noted that EX results in adaptations of many organ systems including adipose tissue and bone which could also contribute to the humoral pathway.

### 4.1. Liver-brain crosstalk

#### 4.1.1. *β*-hydroxybutyrate

The ketone bodies acetoacetate and *β*-hydroxybutyrate (β-HB) serve as fuel substrates that are upregulated in metabolic changes such as caloric restriction (Lin et al., 2015), ketogenic diets, fasting and EX (Sleiman et al., 2016; Evans et al., 2017). It has been reported that β-HB can cross the BBB and function as a signaling molecule promoting BDNF expression after EX (Sleiman et al., 2016). Indeed, these authors demonstrated that hippocampal β-HB contents were significantly increased in mice after 30 days of voluntary wheel running. Furthermore, exposing both cortical neuron cultures and mice hippocampus slices to β-HB led to an overexpression of *bdnf* gene. *In vivo*, intraventricular administration of β-HB in mice induced a significant increase in hippocampal *bdnf* gene expression. Consistently, a positive correlation between plasmatic β-HB levels and hippocampal BDNF contents was highlighted in mice after 6 weeks of voluntary running (Marosi et al., 2016). Interestingly, in the absence of EX, a similar increase in hippocampal BDNF was observed after *in vivo* β-HB administration in mice with normal diet (Hu et al., 2018) while infusion of β-HB attenuates motor deficits in mouse models of Huntington’s disease and protects neurons in models of AD and PD (Kashiwaya et al., 2000). Additionally, a ketogenic diet seems to play neuroprotective roles during cerebral ischemia, neurodegenerative diseases and enhanced memory processes in aged mice (Suzuki et al., 2002; Gano et al., 2014; Paoli et al., 2014; Newman et al., 2017). At the molecular level, β-HB acts directly on *bdnf* gene promoter by HDAC inhibition. Indeed, β-HB exposure of cultured primary neurons decreases HDAC 2/3 activity on the *bdnf* promoter. Besides, the inactivation of HDAC3 by BRD3308 (an inhibitor) or short hairpin RNA strategy, increases *bdnf* transcript expression (Sleiman et al., 2016).

#### 4.1.2. Insulin like growth factor 1

Structurally related to pro-insulin, Insulin like growth factor 1 (IGF-1) is a polypeptide hormone primarily identified in the liver, which can be transported to other tissues and act as an important mediator of body growth and tissue remodelling (Laron, 2001). Although bone and skeletal muscle can produce IGF-1 (Yakar et al., 2010), the major peripheral source of IGF-1 is believed to be the liver as demonstrated by a 75% decrease in plasma concentration in liver-specific IGF-1 deficient mice (Yakar et al., 1999). IGF-1 is involved not only in the growth and development of the brain during early life, but also in its maintenance and plasticity. Once secreted in the systemic circulation, IGF-1 can cross the BBB (Pan and Kastin, 2000) by binding to IGF-1 receptors in a mechanism driven by neuronal activity (Trejo et al., 2001; Nishijima et al., 2010). Data indicated that IGF-1 mediates neurogenesis (Ding et al., 2006), synaptogenesis (O’Kusky et al., 2000), vessels growth (Lopez-Lopez et al., 2004), neuroprotection and neuroplasticity (Ding et al., 2006). In rats, intracarotid injection of IGF-1 induced BDNF mRNA expression in the hippocampus (Carro et al., 2000). In addition, the specific inhibition of IGF-1 receptors in the hippocampus using latex microbeads containing alphaIR3 antibody in trained rats, prevented EX-induced enhancement in memory recall and significantly decreased both pro-BDNF and BDNF expressions (Ding et al., 2006). Similarly, using antiserum raised against IGF-1, Chen and Russo-Neustadt (2007) reported that the blockade of the uptake of peripheral IGF-1 reversed the increase in BDNF mRNA and protein expressions elicited by EX.

#### 4.1.3. Fibroblast growth factor 21

Using liver specific Fibroblast growth factor 21 (FGF21) knockout mice, Markan et al. (2014) demonstrated that FGF21 is preferentially expressed in the liver. FGF21 functions as a metabolic regulator (Kharitonenkov et al., 2005) capable of preventing insulin resistance, increasing fatty acid oxidation (Badman et al., 2007) and weight loss in obese animal models (Jimenez et al., 2018) and humans (Gaich et al., 2013). EX induces an increase in FGF21 expression in mice and humans (Kim et al., 2013) which seems to be dependent on the rise in glucagon-to-insulin ratio since prevention of this increase during EX blunts EX-induced increase in FGF21 (Hansen et al., 2016). In addition to its beneficial effects on the whole body, FGF21 may also exert positive effects on cognition in response to EX. In this regard, FGF21 has been shown to enter the brain (Hsuchou et al., 2007), to improve cognition by restoring synaptic plasticity in obese-insulin-resistant male rats (Sa-Nguanmoo et al., 2016) and to be neuroprotective in a mouse model of aging (Yu et al., 2015). Moreover, data have shown that the intraperitoneal administration of FGF21 in aged mice was associated with activation of the AMPK pathway associated with an increase in cerebral BDNF levels and a significant improvement in Morris water maze assessment (Kang et al., 2020).

#### 4.1.4. Glycosylphosphatidylinositol–specific phospholipase D1 (Gpld1)

Recently, using an elegant plasma transfer strategy, Horowitz et al. (2020) showed that plasma obtained from both aged and mature exercised mice could alleviate hippocampal impairments when transferred to naïve aged mice. Among the 12 factors that were found to be increased in trained mice, the authors focused on Gpld1. They confirmed that this enzyme was predominantly expressed in the liver (Maguire and Gossner, 1995) and that liver Gpld1 expression was increased after EX whereas no change was observed in muscle and hippocampus. Furthermore, Gpld1 plasma contents were significantly enhanced by EX in both mature and aged mice with a significant correlation between liver-derived Gpld1 and cognitive performances in the radial arm water-maze. In addition, they reported that liver overexpression of Gpld1 was associated with a significant increase in hippocampal BDNF expression and an enhancement of neurogenesis markers in the dentate gyrus. These cellular and molecular changes were consistent with an improvement of cognitive function since these mice showed better spatial and recognition memory (Horowitz et al., 2020). Although the precise mechanism underlying the connection between this Glycosylphosphatidylinositol (GPI) hydrolyzing enzyme and the improvement of hippocampal-dependent learning and memory is not fully delineated by the authors, Horowitz and colleagues reported using a catalytically inactive mutant, that the enzymatic activity was necessary for its effects. The authors suggest that this enzyme may be involved in coagulation, as well as in the complement system cascades of molecules downstream of GPI-anchored substrate. Accordingly, in a similar experimental design using plasma transfer from exercising mice, complement and coagulation factor such as clusterin, largely produced by hepatocytes, was recently reported to reduce hippocampal inflammation and promote neurogenesis and cognition although BDNF expression was not assessed in this study (De Miguel et al., 2021).

### 4.2. Muscle-brain crosstalk

Over an extended period, the term “exercise factor” served to depict molecules originating from contracting muscles, potentially orchestrating metabolic and physiological effects on central or peripheral organs. Among these factors, cytokines were the first reported in the literature (Sprenger et al., 1992) and it was through studies showing that muscle IL-6 can act as a humoral factor operating in an autocrine, paracrine, or endocrine manner that the neologism “myokines” was introduced (Pedersen et al., 2003). Although it would have been logical to initiate this muscle-brain crosstalk section with a paragraph dedicated to cytokines, the evidence of a direct impact of these molecules on cerebral BDNF production remains inconclusive despite it has been demonstrated that IL-6 can cross the BBB (Banks et al., 1994). The same lack of direct evidence exists for IL-10 or TNF-α even though studies have reported a similar pattern of variation with BDNF in the hippocampus (Noga et al., 2007; Cabral-Santos et al., 2016; Jahangiri et al., 2019). In contrast, several myokines have been identified, bolstered by compelling scientific rationale, establishing a causative link to brain BDNF expression.

#### 4.2.1. Lactate

Lactate is a metabolite largely produced during EX and mainly released from skeletal muscles. Lactate can cross the BBB via monocarboxylate transporters (MCT) binding on neurogliovascular unit (including astrocytes, neurons, endothelial cells and pericytes) (Proia et al., 2016) and participate to neuroplastic processes such as neurogenesis (Lev-Vachnish et al., 2019), neuronal excitability (Skwarzynska et al., 2023) and LTP (Suzuki et al., 2011). Recently, lactate has been identified as an appealing candidate for inducing cerebral BDNF expression in response to EX. Interestingly, using intraperitoneal infusion of the lactate MCT inhibitor (AR-C155858) in mice submitted to voluntary EX, El Hayek et al. (2019) demonstrated that hippocampal *bdnf* gene expression was completely abolished. Conversely, intraperitoneal administration of lactate in mice elicited a hippocampal BDNF increase similar to that obtained in trained mice. The authors also reported that the improvement in learning/memory performance was dependent on BDNF as co-administration of lactate and a TrkB antagonist (CEP701) prevented this effect. Although the interaction between lactate and cerebral BDNF levels is not fully elucidated, some mechanisms have been proposed [For review (Muller et al., 2020)]. Firstly, lactate promoted plasticity-related gene expressions (*bdnf, Arc, Zif^268^* and *c-fos*) in neuronal cultures of mouse neocortex by potentiating neuronal NMDA glutamatergic transmission (Yang et al., 2014). Secondly, peripheral administration of lactate induced SIRT-1 activity, thereby fostering the cerebral PGC-1α/FNDC5/BDNF pathway (El Hayek et al., 2019). Conversely, inhibition of SIRT-1 with sirtinol administration or RNA interference impeded both hippocampal *bdnf* gene induction and the lactate- and EX-mediated improvement of learning/memory (El Hayek et al., 2019).

#### 4.2.2. PGC-1α pathways

##### 4.2.2.1. FNDC5/irisin

Recent research has provided compelling evidence suggesting that the activation of the PGC-1α FNDC5/irisin pathway in skeletal muscles is also involved in EX-induced cerebral plasticity (Lourenco et al., 2019). Using AD mice models, the authors demonstrated that FNDC5 adenovirus delivery through the tail vein or intra-cerebrovascular injection of FNDC5 rescued memory impairment and synaptic plasticity mechanisms dependent on cerebral BDNF. Conversely, the blockade of peripheral FNDC5/irisin attenuates the effect of EX on LTP and memory tests. Although this blockade was not directly linked to a decrease in brain BDNF expression in this study, these data support the role of peripheral FNDC5/irisin on physiological memory process. In this regard, in the context of hypertension, EX-induced enhanced BDNF expression was reported to be dependent on peripheral but not central FNDC5 (Wang et al., 2019). Furthermore, based on the premise that irisin is secreted by the skeletal muscle, Islam et al. (2021) have provided evidence that irisin can cross the BBB. Indeed, through the injection of fluorescently labeled irisin into the bloodstream of mice and subsequent confocal microscopy analysis, the authors successfully detected the distribution of labeled irisin within the cerebral region. Then, they have shown that peripheral delivery of irisin improved learning and memory in young and old mice and significantly reduced the buildup of beta-amyloid plaques in a mouse model of AD. Collectively, these findings support the hypothesis that FNDC5/irisin may cross the BBB or induce another factor that triggers cerebral BDNF expression, possibly through CREB activation, as previously stated. Accordingly, a very recent study reported that irisin binds to activated integrin αVβ5 receptors, which are abundantly expressed on brain endothelial cells (Mu et al., 2023). However, while FNDC5/irisin appears as a very attractive myokine, it is important to note that the detection of this hormone by commercial antibodies reveals an important cross-reactivity (Albrecht et al., 2015). Besides, the translatability of the studies from animals to humans may encounter a major pitfall since the transcription pattern of *fndc5* gene is not conserved from rodent to human resulting in very low translation efficiency (Raschke et al., 2013; Albrecht et al., 2020).

##### 4.2.2.2. Kynurenine to kynurenic acid pathway

Another PGC-1α dependent mechanism involves the conversion of Kynurenine (KYN) to kynurenic acid (KYNA). Indeed, tryptophan, primarily derived from the liver, is metabolized to KYN through indolamine-2,3-dioxygenase (IDO) (Martin et al., 2020). Interestingly, KYN has been reported to cross the BBB whereas KYNA cannot (Fukui et al., 1991) and was shown to be neurotoxic (Sundaram et al., 2020) leading to depression and neurodegenerative disorders (Myint et al., 2007). Conversely, KYNA which is produced from KYN via muscle kynurenine aminotransferases (KAT) activity (Schwarcz et al., 2012), is neuroprotective (Foster et al., 1984) and cognitive performance were positively associated with KYNA concentrations in plasma of AD patients (Gulaj et al., 2010). Therefore, maintaining a well-balanced ratio between these two metabolites appears to be crucial. Following EX, the activation of the PGC-1α pathway stimulates the expression of KAT enzymes within skeletal muscle. Consequently, data also reported that EX increases plasma KYNA levels both in rodents (Agudelo et al., 2014) and humans (Lewis et al., 2010) while an increase in KYN was shown in a model of stress-induced depression (Agudelo et al., 2014). Interestingly, mice with muscle-specific overexpression of PGC-1α were resilient to stress-induced depression compared to wild-type littermates and did not exhibit decreased hippocampal *bdnf* gene expression while peripheral KYN administration induced depressive behavior in wild-type but not in transgenic animals (Agudelo et al., 2014). Consistent with these results, in a murine model of PD, IDO inhibition was associated with reduced oxidative stress, lower impairment in coordination and locomotion and restoration of striatal BDNF levels (Sodhi et al., 2021). Collectively, these data emphasize the importance of muscle KYN-to-KYNA conversion in facilitating BDNF-induced cognitive improvement after EX.

#### 4.2.3. Cathepsin-B

Cathepsin-B (CTSB), a lysosomal cysteine protease ubiquitously expressed was identified in 2016 as a myokine by Moon et al. (2016). Using proteomic analysis of the culture media from L6 myoblast cells treated with the AMPK agonist 5-aminoimidazole-4-carboxamide ribonucleotide (AICAR) to mimic the effects of EX, the authors reported an increase in CTSB. To validate CTSB as a candidate myokine, they further showed that the rise in plasma CTSB levels in voluntary running mice was concomitant with an increase in CTSB mRNA and protein expressions in gastrocnemius muscles after 30 days. In addition, similar increases in CTSB plasma levels were observed in Rhesus monkeys and humans after four months of treadmill running. To delineate the underlying mechanisms, Moon et al. (2016) conducted studies in CTSB KO mice showing that compared to wild-type control mice, EX in CTSB KO mice failed to enhance neurogenesis and improve spatial memory. Additionally, intravenous injection of CTSB in CTSB KO mice led to a significant increase in CTSB levels in the blood and brain tissues, indicating that CTSB can cross the BBB. Interestingly, these authors demonstrated that recombinant CTSB administration induced the increase in both doublecortin and BDNF mRNA levels in adult hippocampal progenitor cells. These data are consistent with a previous study showing in contrast that inhibition of cathepsins B and L reduced kainate-induced BDNF mRNA expression in cultured hippocampal slices (Bednarski et al., 1998). Although these data support a causative link between CTSB and cerebral BDNF expressions, the complex interplay between them should be interpreted considering the origin of CTSB and the specific physiological context. Indeed, recent studies have suggested that microglia-derived CTSB may be a key driver in inflammatory brain diseases and aging (Nakanishi, 2020) while genetic CTSB deletion prevented cognitive impairments, reduced amyloid peptides, brain damage and pro-inflammatory factors, in animal models of traumatic brain injury, AD and aging (Hook et al., 2022).

#### 4.2.4. BDNF as a myokine?

BDNF is produced in skeletal muscle by various cell types, including myofibers, satellite cells, motoneurons and endothelial cells (Mousavi and Jasmin, 2006; Matthews et al., 2009; Cefis et al., 2022). BDNF has been found to play an important role in muscle growth, function, regeneration (Mousavi and Jasmin, 2006; Clow and Jasmin, 2010) and metabolism (Matthews et al., 2009; Yang et al., 2019). In response to EX, a significant increase in BDNF mRNA and/or protein levels has been observed in skeletal muscles (Cuppini et al., 2007; Ogborn and Gardiner, 2010; Yu et al., 2017). According to the definition of myokines, BDNF could be classified as one (Matthews et al., 2009; Yang et al., 2019). However, whether it may be tempting to believe that skeletal muscle could be a primary source of circulating BDNF levels remains a subject of debate. Although the muscle cells produce BDNF, the precise mechanism of its release into the bloodstream remains uncertain, and there is supportive evidence from various studies indicating that circulating BDNF predominantly originates from the brain (Rasmussen et al., 2009). Thus, using prolonged EX, Rasmussen and collaborators demonstrated in Human that the increase in circulating BDNF originated from the brain, as evidenced by the difference between plasmatic BDNF from arterial and jugular origins (Rasmussen et al., 2009). Besides, in skeletal muscle, a study using electroporation to overproduce BDNF in mice failed to increase circulating BDNF levels, suggesting that muscle derived-BDNF may act only in an auto/paracrine manner (Matthews et al., 2009). However, recent evidences have shown that neuromuscular electric stimulation (NMES) increases circulating BDNF levels both in humans (Miyamoto et al., 2018; Kimura et al., 2019) and animals (Fulgenzi et al., 2020). Additionally, it has been recently demonstrated for the first time that human myocytes produced and secreted biologically active BDNF (Fulgenzi et al., 2020). These outcomes reinforce the idea that skeletal muscle could be a potential source of circulating BDNF in humans and animals, but a further question still needs to be clarified: Is BDNF released from the skeletal muscle into bloodstream can directly interact with the brain?

To address this question, particular attention should be provided to extracellular vesicles, especially exosomes. Based on the provided references, BDNF can be transported within exosomes, (Yuan et al., 2017; Gelle et al., 2021) and it has been observed that muscle contraction induced their release (Watanabe et al., 2022). At the cerebral level, it was recently suggested that BDNF transported in exosomes could allow sustained and specific release of BDNF in the brain (Yuan et al., 2017), a mechanism that could potentially extend to BDNF derived from skeletal muscle. In addition, the transport by exosomes offers a considerable advantage since exosomes can cross the BBB (Yuan et al., 2017; D’Anca et al., 2019). Indeed, at the peripheral level, exosomes could allow BDNF, which has a short half-life, to evade the catabolic processes. Finally, even if modulation of neuroplasticity by skeletal muscle-derived BDNF has yet to be demonstrated, peripheral delivery of BDNF has been shown to induce neurogenesis and increase BDNF levels in hippocampus, leading to antidepressant and anxiolytic effects in mice (Schmidt and Duman, 2010). Taken together, these findings support the hypothesis that muscle-derived BDNF, may cross the BBB and positively act in the brain following EX. Further *in vivo* studies are needed to provide more conclusive evidence in support of this hypothesis.

## 5. Conclusion and future directions

As the most downstream factor mediating EX-induced brain health, manipulating BDNF content in the brain has emerged as a promising strategy for mitigating cognitive deficits, addressing neurodegenerative disorders, counteracting age-related cognitive impairment, and promoting overall brain health. EX has been identified as a potent and robust non-pharmacological intervention for enhancing cognitive function and limiting cognitive deficits through increased brain BDNF levels. Recent research has shed light on two additional pathways, namely hemodynamic and humoral, which complement the well-established role of neuronal activity in modulating brain BDNF levels. Notably, these pathways offer more feasible and achievable pharmacological strategies for intensifying brain BDNF expression compared to the neuronal activity pathway, as they circumvent the challenges of drug delivery across the BBB and potential drug-related side effects on neurotransmission.

However, the question of the relative contribution of each of these mechanisms to the beneficial effects of EX-induced brain BDNF increase remains complex and challenging to answer definitively. It is hypothesized that each pathway plays an essential role, as strategies that dampen any of these mechanisms individually have been shown to result in defective BDNF expression associated with cognitive impairment. It is plausible that these distinct mechanisms exist to offer a differential and persistent age-dependent temporal response, or to compensate for the deficiencies of one pathway with the others. Moreover, evidence suggests that these mechanisms are intricately interconnected, as neuronal activation is coupled with CBF regulation, and irisin, one of the well-studied humoral factors, has been found to modulate both vasorelaxation and neuronal activity. To understand the contributive part of the different pathways involved, employing transgenic mice with selective endothelial deletion of tPA or BDNF could provide valuable insights into the neuroplastic consequences of the hemodynamic pathway. Similarly, conducting studies that compare NMES to conventional models of EX would enable the isolation of the humoral muscle-brain crosstalk from the broader whole-body response. Indeed, this strategy would overcome an experimental bias as current studies investigating the muscle-brain dialogue are conducted following conventional protocols which engender a pre-dialogue between peripheral tissues that are also responsive to EX. The conversion of KYN into KYNA or the production of irisin by adipose tissue are some examples.

In conclusion, manipulating brain BDNF content through non-pharmacological interventions such as EX has emerged as a promising strategy to enhance cognitive function and mitigate cognitive deficits. Recent findings on hemodynamic and humoral pathways provide additional insights into the mechanisms through which EX induces brain BDNF overproduction. Further research is warranted to better understand the contribution of each pathway to the beneficial effects of EX on brain health and cognitive function. In addition, due to a wide range of EX types and the complexity of their classification, it is crucial to keep in mind that the contribution of the pathways described could be contingent upon EX type (e.g., resistance, aerobic), duration, intensity, frequency. Although this aspect has not been addressed in the manuscript, we encourage readers to consider this remark. Altogether, the findings on hemodynamic and humoral pathways will undoubtedly have implications for clinicians aiming to promote EX interventions or for scientists seeking to develop alternative strategies to mimic EX effects, particularly in populations who may face challenges with regular EX adherence. By unraveling the intricate interplay of these mechanisms, the scientific community can unlock the full potential of brain BDNF modulation for cognitive health and neuroprotection in various clinical and research settings.

## Author contributions

MC: Writing – original draft, Writing – review & editing. RC: Writing – original draft, Writing – review & editing. JW: Writing – review & editing. AM: Writing – review & editing. AQ: Writing – review & editing. CL: Writing – review & editing. AP-T: Writing – original draft, Writing – review & editing. PG: Writing – original draft, Writing – review & editing.
